# Cost-effectiveness of QuantiFERON for TB infection screening in household contacts in the Philippines

**DOI:** 10.5588/ijtldopen.26.0265

**Published:** 2026-09-15

**Authors:** L.L. Mortera, J.O. Padua, R. Alagna

**Affiliations:** 1 Lung Center of the Philippines, Manila, Philippines;; 2 University of Santo Tomas Hospital, Manila, Philippines;; 3 QIAGEN S.r.l., Milan, Italy.

**Keywords:** tuberculosis, TBI, diagnostics, cost-effectiveness, household contacts

Dear Editor,

TB is still a clinical and public health priority in the Philippines, one of the 30 top high-burden countries, where the WHO estimated incidence was 643/100,000 and the mortality 32/100,000 population.^1^ Approximately one in four individuals in the world is infected by *Mycobacterium tuberculosis* and 5%–15% of them, if not treated, develop active TB during their life.^2,3^ The WHO End TB Strategy aims to eliminate TB as a public health threat by 2030.^4^ Detecting and treating TB infection (TBI) among high-risk populations (including contacts of infectious TB patients) is essential to prevent future TB cases to occur.^5^ National TB programmes in the Philippines and in other high-burden and limited-resource countries need to implement cost-effective TBI screening strategies to identify infected individuals and treat them adequately.^6,7^

To inform national TB control policy and support the implementation of targeted interventions contributing to TB elimination goals in the Philippines, this study evaluates the cost-effectiveness of TBI screening of household contacts based on QuantiFERON versus the tuberculin skin test (TST) by assessing the economic and health outcomes of each strategy. A cohort-based model was developed to estimate the costs and health outcomes of QuantiFERON in comparison to TST for the household contacts population in the Philippines from a health care perspective.^8^ The model included a decision tree capturing outcomes related to TBI screening and a cohort Markov model simulating the lifetime costs and benefits of the patient cohort. The model was not designed to capture the impacts of secondary infections. It was assumed that the cohorts were screened for active TB and all current active cases were identified. The modelling approach was developed with the guidance of an advisory board.^8^ The economic model was built using R version 4.0.2 with an interactive R shiny interface. The model was designed in a flexible and user-friendly format using simple point and click functionality where appropriate (e.g., to run probabilistic sensitivity analysis). A fully incremental approach was taken to the analysis: all interventions were assessed simultaneously rather than making pairwise comparisons between QuantiFERON and TST. Inputs were derived from published literature (see references in the Annexes), expert opinion, and available country-level data. Key outcomes included costs, quality-adjusted life years (QALYs), and incremental cost-effectiveness ratios. Deterministic and probabilistic sensitivity analyses were conducted, along with scenario analyses to explore the impact of varying TBI prevalence, reactivation risk, and population characteristics (Supplementary Data).

The case analysis indicates that QuantiFERON performs better than TST: 1) being less costly and slightly more effective than TST, 2) allowing cost savings of $29.81, and 3) 0.01 QALYs gain per patient (Table 1). As shown in Table 2, QuantiFERON also yields a marginally higher number of discounted life years per patient (24.6436 vs. 24.6424). These findings support the conclusion that QuantiFERON is cost-effective when compared with TST. Table 3 reports the discounted cost breakdown per patient associated with QuantiFERON and TST. In Table 4, the predicted lifetime likelihood of developing active drug-susceptible or drug-resistant TB on a per-patient basis is shown, being 0.52% and 0.84%, respectively, for QuantiFERON and TST, with a 0.32% gain in favour of QuantiFERON. Based on this difference, the model estimates that testing 311 individuals with QuantiFERON rather than TST would prevent one additional case of active TB. Furthermore, the cost per TB case avoided is –$9,279, indicating that QuantiFERON not only prevents more cases but also leads to cost savings of $9,279 for each additional TB case prevented.

**Table 1.** Summary of the cost-effectiveness results of QuantiFERON and tuberculin skin test (TST): base case results per patient.

Strategy	Cost	QALYs	Change in costs	Change in QALYs	ICER (cost per QALY gained)
QuantiFERON	$184.30	25.63			
TST	$214.11	25.61	$29.81	−0.01	Dominated

$ = United States dollar; QALY = quality-adjusted life year; ICER = incremental cost-effectiveness ratio.

**Table 2.** Life years gained per patient.

Item	QuantiFERON	TST
Discounted life years	24.6436	24.6424
Incremental		0.0012

TST = tuberculin skin test.

**Table 3.** Summary of the cost-effectiveness results of QuantiFERON and tuberculin skin test (TST): discounted cost breakdown per patient.

Item	QuantiFERON	TST
Screening	$56	$16.80
Treatment	$5.61	$8.61
Health care worker contact time	$29.3	$44.41
TBI adverse events	$25.02	$38.31
Diagnosis tests	$1.15	$1.87
Active TB case contacts	$10.69	$17.30
Other	$56.78	$86.83
Total	$184.30	$214.11

TBI = TB infection.

**Table 4.** Summary of the cost-effectiveness results of QuantiFERON and tuberculin skin test (TST): active TB cases avoided.

Item	QuantiFERON	TST
Life time active TB rate	0.0052	0.0084
Incremental cost		−0.0032
Cost per avoided active TB case		−$9,279
Number needed to treat		311

The findings show that QuantiFERON is more effective and less costly than TST. In particular, QuantiFERON allowed per-patient cost saving of $29.81 with an incremental gain of 0.01 QALYs. In terms of life expectancy, QuantiFERON provided a marginally higher number of discounted life years per patient (24.6436 vs. 24.6424), reinforcing its clinical and economic value. Importantly, QuantiFERON was also associated with a reduced lifetime risk of developing active TB compared to TST (0.52% vs. 0.84%), corresponding to a 0.32% absolute risk reduction. The model estimates that testing 311 individuals with QuantiFERON rather than TST would prevent one additional TB case. The negative cost per avoided TB case (–$9,279) indicates that QuantiFERON prevents additional TB cases while producing overall cost savings to the health care system.

The deterministic sensitivity analysis showed that the model’s conclusions were robust across a wide range of input parameters (see Supplementary Data). The top drivers influencing the cost-effectiveness results were the discount rate for health benefits, the average age of the cohort, the proportion of men, and the utility value assigned to TBI without adverse events. Furthermore, varying these inputs by ±15% did not change the direction of the results, suggesting that QuantiFERON consistently remains the more cost-effective option compared to TST. Similarly, the probabilistic sensitivity analysis confirmed that QuantiFERON is more cost-effective than TST in the majority of simulated scenarios. Although a small number of simulations suggested that TST could be slightly cheaper, QuantiFERON consistently demonstrated greater effectiveness. The clustering of QuantiFERON scenarios around lower cost and higher QALYs, and its favourable average position on the cost-effectiveness plane, further support its economic advantage. Although the differences in QALYs and life years between QuantiFERON and TST are small on a per-patient basis, these differences are important when scaled to large populations. Additionally, the reduced TB risk and lower associated treatment costs with QuantiFERON support its public health relevance, particularly in the perspective of TB elimination.^7–9^

From a policy perspective, the results support the use of QuantiFERON as a preferred screening tool over TST in household contact of Philippines, aligning with WHO recommendations (e.g., targeted TBI screening for high-risk groups).^5^ Given the added advantages of single-visit testing, reduced reader variability, and lower risk of false positives due to bacille Calmette-Guérin (BCG) vaccination, QuantiFERON offers both clinical and operational benefits.^10,11^

In conclusion, this study provides robust evidence that QuantiFERON is more cost-effective than TST for TBI screening in the Philippines household contact population. It achieves modest improvements in health outcomes at a lower overall cost and contributes to reducing the future burden of active TB. These findings support broader adoption of QuantiFERON in national TB screening strategies, particularly in high-risk, resource-constrained settings like the Philippines where efficiency and impact must be balanced.

## Supplementary Material
