# Supplementary material for: Cost-effectiveness of QuantiFERON for TB infection screening in household contacts in the Philippines
Source: IJTLD Open. 2026 Sep 15;3(9):601–3. doi: 10.5588/ijtldopen.26.0265 (PMC13577214; doi:10.5588/ijtldopen.26.0265)
Supplement: Supplementary file 1 [file ijtldopen26-0265_supplementarydata1.pdf]

Electronic Annex (EA)

The key elements of the modelling approach adopted are summarised in EA1.

Electronic Annex 1. Summary of the decision problem

Model element	Description
Intervention	QuantiFERON
Comparator(s)	TST
Population (base case)	Household contacts into Philippines
Perspective on costs	Healthcare payer
Time horizon	Lifetime
Cycle length	1 year
Discount rate (benefits)	3% per year
Discount rate (costs)	3% per year
Cost effectiveness threshold	$1,105 USD per QALY gained

The decision tree structure is demonstrated in EA2.

Electronic Annex 2. Decision tree structure

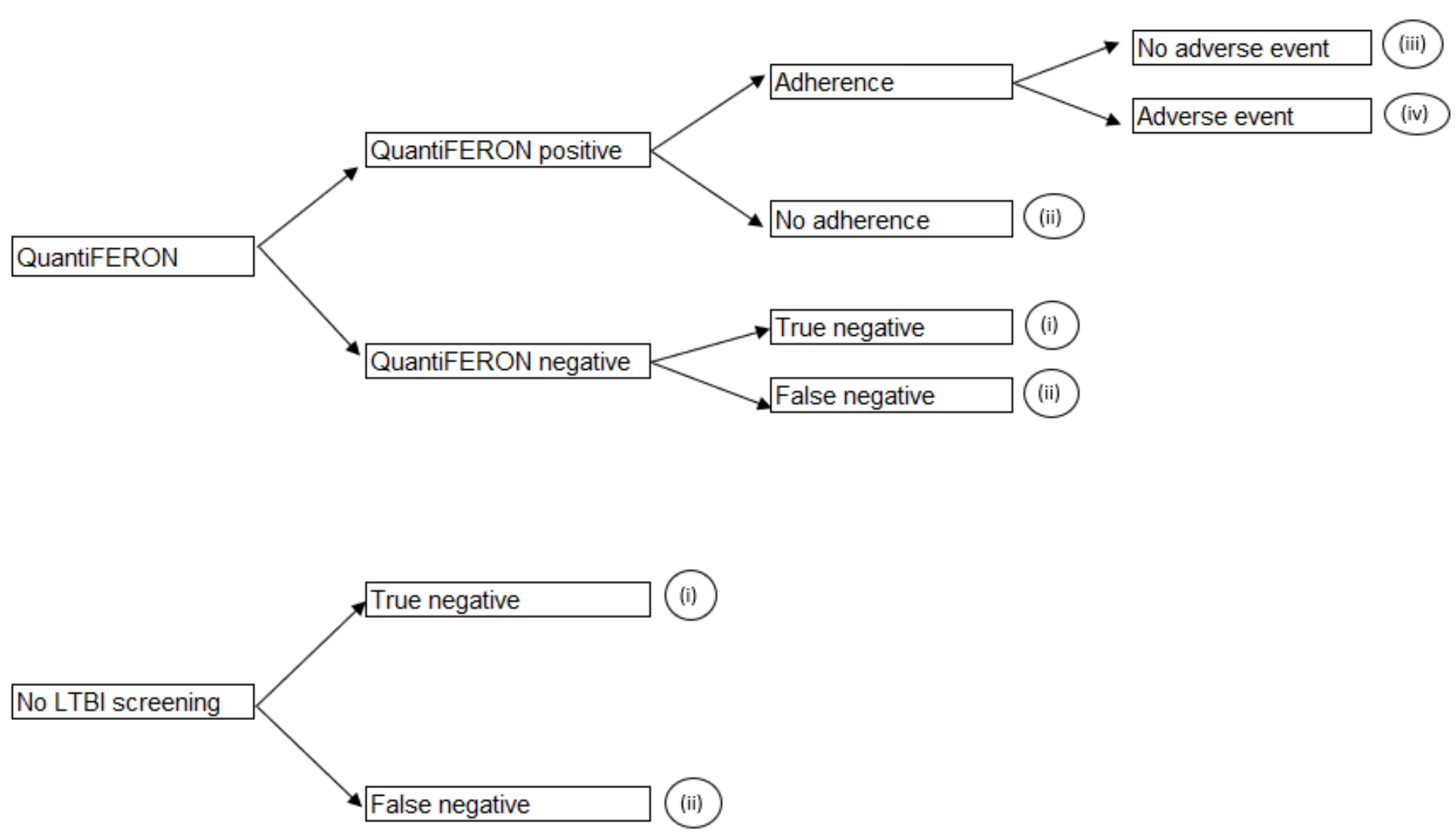

The four health states leaving the decision tree i) Healthy ii) Untreated TBI (treatment naïve) iii) TBI, taking treatment without adverse events and iv) TBI, taking treatment with adverse events.

The Markov model was made up of the following eight mutually exclusive health states shown EA3.

Electronic Annex 3. Markov model health states

Health state	Description
Healthy	Person without TBI.
Untreated TBI (treatment naïve)	Person with TBI but who was not diagnosed, or was diagnosed with TBI but did not initiate treatment. This person is at risk of developing active TB.
TBI, taking treatment without adverse events (AE)	Person diagnosed with TBI and taking treatment. The person does not experience AE relating to TBI treatment.
TBI taking treatment with AE	Person diagnosed with TBI and taking treatment. The person experiences AE relating to TBI treatment.
Untreated TBI (previously treated)	Person who was previously diagnosed with TBI and received treatment. However, TBI was not successfully cleared, and the person remains at risk of developing active TB (lower risk compared with the TBI treatment naïve group).
Drug sensitive TB (DS TB)	Person diagnosed with drug-sensitive TB.
Multidrug resistant TB (MDR TB)	Person diagnosed with multidrug resistant TB.

These health states were chosen to align with previous modelling of TB screening tests as well as incorporate clinical expert feedback. The Markov model used a lifetime time horizon and a cycle length of 1 year, consistent with existing models in this area. EA4 shows the transitions between states of the Markov model.

Electronic Annex 4. Markov model structure

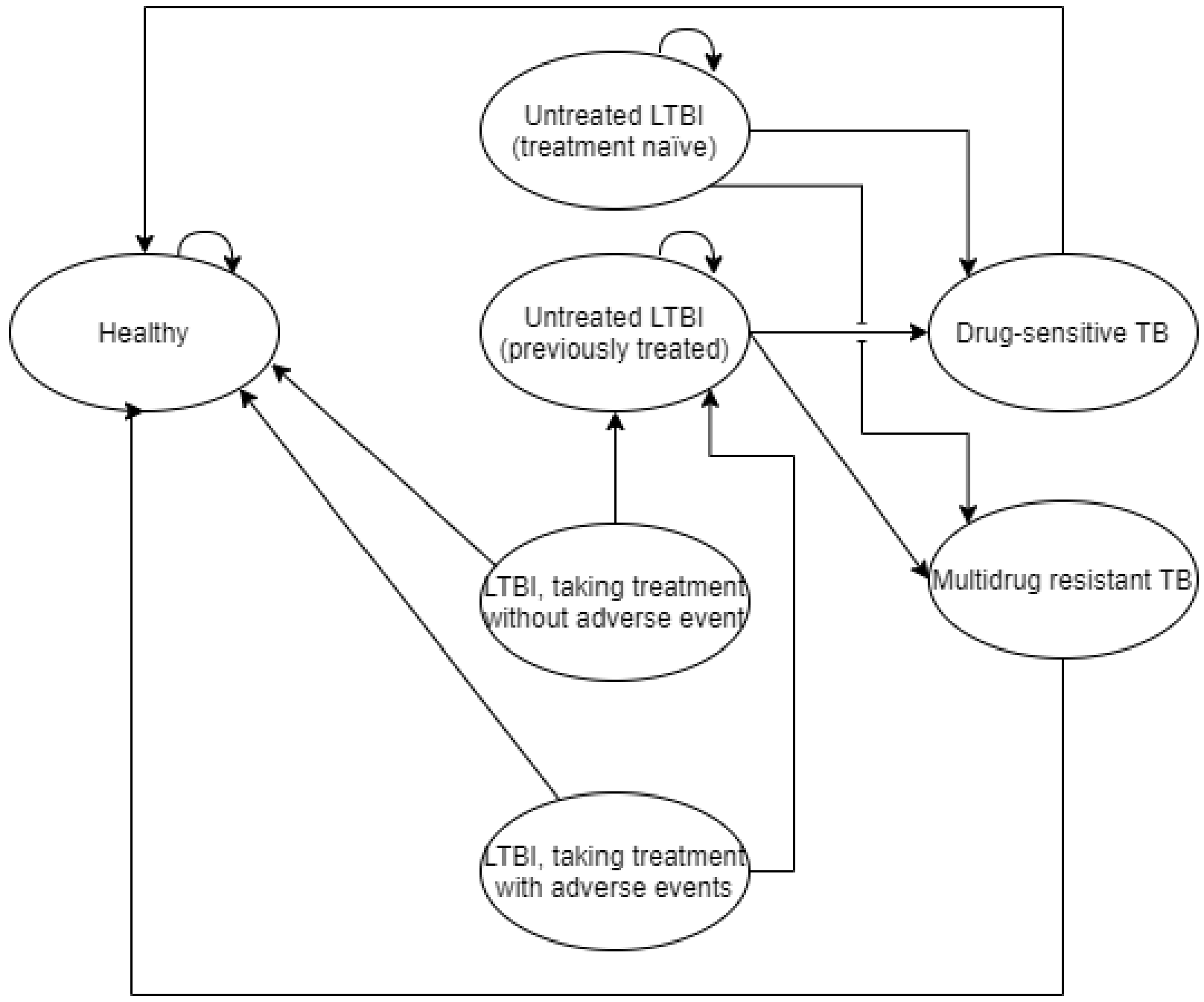

Transitions between Markov health states were determined by a set of transition probabilities. A patient in the healthy state would always remain in the healthy state as the model was not designed to consider secondary infections or subsequent screening processes. Patients treated for TBI (with or without AE) either returned to full health or transitioned into the untreated TBI (previously treated) state.

Patients with untreated TBI (treatment naïve or previously treated) either remained untreated or transitioned into an active TB state (DS TB or MDR TB). Because the probability of reactivation from TBI decreases over time, TBI reactivation curves were used to estimate the reducing probability of TBI reactivation each cycle.

It was assumed that all patients treated for active TB were cured with no further risk of TB reactivation. Transitions into the dead state were permitted from all health states.

The methods of derivation are presented EA5 (transitions to the dead state not presented)

Electronic Annex 5. Transition probabilities used in the first year of the Markov model

* Values were updated every cycle. See Section **Errore. L'origine riferimento non è stata trovata.** for further details.

To / From	(i) Healthy	(ii) Untreated TBI (treatment naïve)	(iii) TBI, taking treatment without AE	(iv) TBI, taking treatment with AE	(v) Untreated TBI (previously treated)	(vi) Drug-sensitive TB being treated	(vii) Multidrug resistant TB being treated
(i) Healthy	100%	0%	0%	0%	0%	0%	0%
(ii) Untreated TBI (treatment naïve)	0%	Residual*	0%	0%	0%	Calculated from TBI reactivation rates*	Calculated from TBI reactivation rates*
(iii) TBI, taking treatment without AE	Calculated from treatment effectiveness	0%	0%	0%	Calculated from treatment effectiveness	0%	0%
(iv) TBI, taking treatment with AE	Calculated from treatment effectiveness	0%	0%	0%	Calculated from treatment effectiveness	0%	0%
(v) Untreated TBI (previously treated)	0%	0%	0%	0%	Residual*	Calculated from TBI reactivation rates*	Calculated from TBI reactivation rates*
(vi) Drug-sensitive TB being treated	100%	0%	0%	0%	0%	0%	0%
(vii) Multidrug resistant TB being treated	100%	0%	0%	0%	0%	0%	0%

Incremental cost-effectiveness ratio

The total costs and QALYs for each screening strategy over a lifetime time horizon were presented. In order to measure the cost-effectiveness of a chosen intervention it was necessary to compare the costs and QALYs generated by each screening strategy. This comparison was facilitated by the calculation of the incremental cost-effectiveness ratio (ICER). The ICER represents the economic value of an intervention, compared with an alternative (comparator) and was calculated for each comparator as:

$$ICER = \frac{Cost_{intervention} - Cost_{comparator}}{QALY_{intervention} - QALY_{comparator}}$$

To determine which screening method was the most cost-effective, a fully incremental analysis was conducted. Fully incremental analysis involves the calculation of incremental QALY gains and costs along a list of interventions ranked by ascending cost. In fully incremental analysis, each comparator is compared with the previous non-dominated alternative. A dominated

intervention is both more costly and less effective than the comparator. A dominant intervention is both less costly and more effective than the comparator.

The resulting ICERs were compared against a pre-established cost-effectiveness threshold in order to establish whether a screening strategy was an efficient use of healthcare resources. An explicit cost-effectiveness threshold is not used for health care decision making in Philippines. However, a cost-effectiveness threshold of $1,105 USD was used in this analysis, taken from a valuation study to determine the cost-effectiveness threshold for health care interventions in Philippines.

Both costs and benefits were discounted at an annual rate of 3%.

Life years gained

Life years gained is a measure of mortality where the remaining life expectancy from the point the patient cohort enter the model is taken into account (i.e. the additional number of years a patient lives as a result of the chosen intervention). The life years gained were presented as discounted life years per patient stratified by screening arm as well as the incremental difference between each screening arm compared with QuantiFERON.

Active tuberculosis cases avoid

The lifetime rate of developing active TB (DS and MDR) per patient in the entire cohort was presented as well as the incremental difference between each screening arm compared with QuantiFERON. The incremental differences in lifetime TB rates, in addition to the incremental costs, were used to calculate the additional costs associated with treating one active TB case compared with QuantiFERON. The number needed to treat (NNT) to avoid one additional case of active TB using QuantiFERON compared with the other screening options were also presented. A negative NNT value represents the number needed to harm (NNH), where the number represents the number of people needed to be screened to result in one additional case of TB.

Model inputs

The model inputs used to run the model are summarised in EA6. These inputs were obtained either from published literature or national sources. Where country-specific data were not available, inputs were retrieved from countries with similar contexts.

Electronic Annex 6. Model inputs

Parameter	Value	Standard Error	PSA Distribution	References
Population size	1.842.464	N/A	N/A	WHO Tuberculosis profile: Philippines (1)
Average age	25.7	N/A	N/A	Worldometer. Philippines demographics (2)
Proportion male	0.49	N/A	N/A	Putra IWGAE, et al. (3)
Active TB prevalence	0.128	0.0128		Sia IG, et al. (4)
TBI prevalence	0.37	0.037	Beta	Ding C, et al. (5)
All-cause mortality	Age-dependant	N/A	N/A	WHO, 2024 (6)
DS TB mortality risk	0.05	0.005	Beta	Chung-Delgado K, et al. (7)
MDR TB mortality risk	0.375	0.0375	Beta	Chung-Delgado K, et al. (7)
Treatment efficacy	0.69	0.069	Beta	International Union Against Tuberculosis (8)
Adherence rate	0.89	0.089	Beta	Calderon JS, et al. (9)
Probability of AE (drug-related hepatotoxicity)	0.36	0.036	Beta	World Health Organization. WHO TB Knowledge Sharing Platform: Module 2 (10)
Initial reactivation rate (treatment naive)	0.0281	0.0033	Beta	Gupta et al., (11)
Initial reactivation rate (previously treated)	0.061	0.0073	Beta	Derived from Gupta et al. (11)
Proportion of MDR TB cases (treatment naive)	0.02	0.002	Beta	Lofranco VS, et al. (12)

Proportion of MDR TB cases (previously treated)	0.166	0.0166	Beta	
Proportion BCG-vaccinated	0.91	N/A	N/A	World Health Organization. BCG vaccination coverage: Philippines. (13)
Sensitivity of TST	0.77	0.03	Beta	Pai et al., (14)
Specificity of TST (BCG-vaccinated)	0.59	0.07	Beta	
Specificity of TST (non BCG-vaccinated)	0.97	0.03	Beta	
Sensitivity of QuantiFERON	0.91	0.03	Beta	Sotgiu et al (15)
Specificity of QuantiFERON	0.95	0.01	Beta	
Utility values				
Healthy	1	N/A	N/A	Dion et al., (16); Guo et al., (17)
Untreated TBI	1	N/A	N/A	
TBI, taking treatment without AE	0.99	0.099	Beta	
TBI, taking treatment with AE	0.85	0.085	Beta	
DS TB	0.8	0.08	Beta	
MDR TB	0.58	0.058	Beta	

Costs and resources

Resource use data were combined with unit costs to determine the total estimated costs for each screening arm in the model. Within the decision tree, each branch is associated with a set of resources which were consumed precisely once. For example, the TST branch consumed one TST only.

The resource use associated with each Markov health state were estimated per cycle, and patients continued to consume these resources for as long as they spend time in the health state. Unit costs and resource use were sourced from national sources for Philippines. The resource use are summarised in EA7 and unit costs in EA8.

Electronic Annex 7.   Resource use

Resource	Time (hours) or #
**TBI, taking treatment without AE**	
Nurse time	1.75
Hospital physician time	3.5
General practitioner	3.5
DOT sessions	7
**TBI, taking treatment with AE**	
Nurse time	3.5
Hospital physician time	3.5
General practitioner	3.5
DOT sessions	7
Liver Function test	3
Lab time for liver function test	4
Treatment of drug induced hepatotoxicity	14
**Drug sensitive TB being treated**	
Nurse time	2.9
Hospital physician time	4.65
General practitioner	4.65
TB specialist time	0.345
DOT sessions	9.3
Smear test of sputum	6.15
Culture of sputum examination	0
Chest x-ray	2.46
Laboratory time for diagnosis tests	4.92
GeneXpert	1
Contacts per active case	4
Hospitalisation	5.15
**Multidrug resistant TB being treated**	
Nurse time	5.25
Hospital physician time	5.6
General practitioner	5.6
TB specialist time	1.9
DOT sessions	15
Smear test of sputum	12.8

Culture of sputum examination	9.6
Chest x-ray	3.2
Laboratory time for diagnosis tests	6.4
GeneXpert	1
Contacts per active case	4
Hospitalisation	7

Electronic Annex 8. Unit costs

Parameter	Description	Cost (USD)
TBI treatment	The average cost of TBI treatment per patient.	17.4
Drug-sensitive TB treatment	The average cost of treating drug-sensitive TB per patient.	104.8
Multi drug resistant TB treatment	The average cost of treating multi drug resistant TB per patient.	424.9
QuantiFERON	The cost of a QuantiFERON test for one patient.	47.7
TST	The cost of a TST for one patient.	14.8
**Healthcare worker contact time**		
Nurse time	The cost of a nurse per hour.	4
Hospital physician time	The cost of a hospital physician per hour.	8.5
General practitioner	The cost of a general practitioner per hour.	8.5
TB specialist time	The cost of a TB specialist per hour.	8.5
DOT sessions	The cost of a DOT session.	4
Environmental health officer	The cost of an environmental health officer per hour	4
**Diagnostic tests**		
Smear test of sputum	The cost of a smear tests per patient.	8.7
Culture of sputum examination	The cost of a culture tests per patient.	65.4
Polymerase chain reaction (PCR) test	The cost of a PCR tests per patient.	126.4
Chest x-ray	The cost of a chest x-rays per patient.	8.7
Laboratory technician time	The cost of a laboratory technician per hour.	2.1
**Drug resistance tests**		
Laboratory technician time	The cost of a laboratory technician per hour.	2.1
**TBI adverse events**		
Liver function test	The number of liver function tests per patient.	5.2
Laboratory technician time	The cost of a laboratory technician per hour.	2.1
Treatment of drug-induced hepatotoxicity	The average cost of treatments per patient.	14.6
**Active TB case contacts**		
Contacts per active case	The average cost associated with contacts per active case of TB.	645.1
**Other**		
Hospitalisation	The average cost of hospitalisation per day.	43.6

Sensitivity analysis

For all included parameters, reasonable higher and lower values were applied around the point estimate (15% above and below) that was used in the base case analysis to highlight the parameters that the model results are most sensitive to. EA9 shows the top 10 drivers of the results when all else remains constant with the 4 biggest drivers including discount rate for benefits, cohort age, proportion male and TBI (no adverse event) utility value.

Electronic Annex 9. Top 10 drivers of the economic model

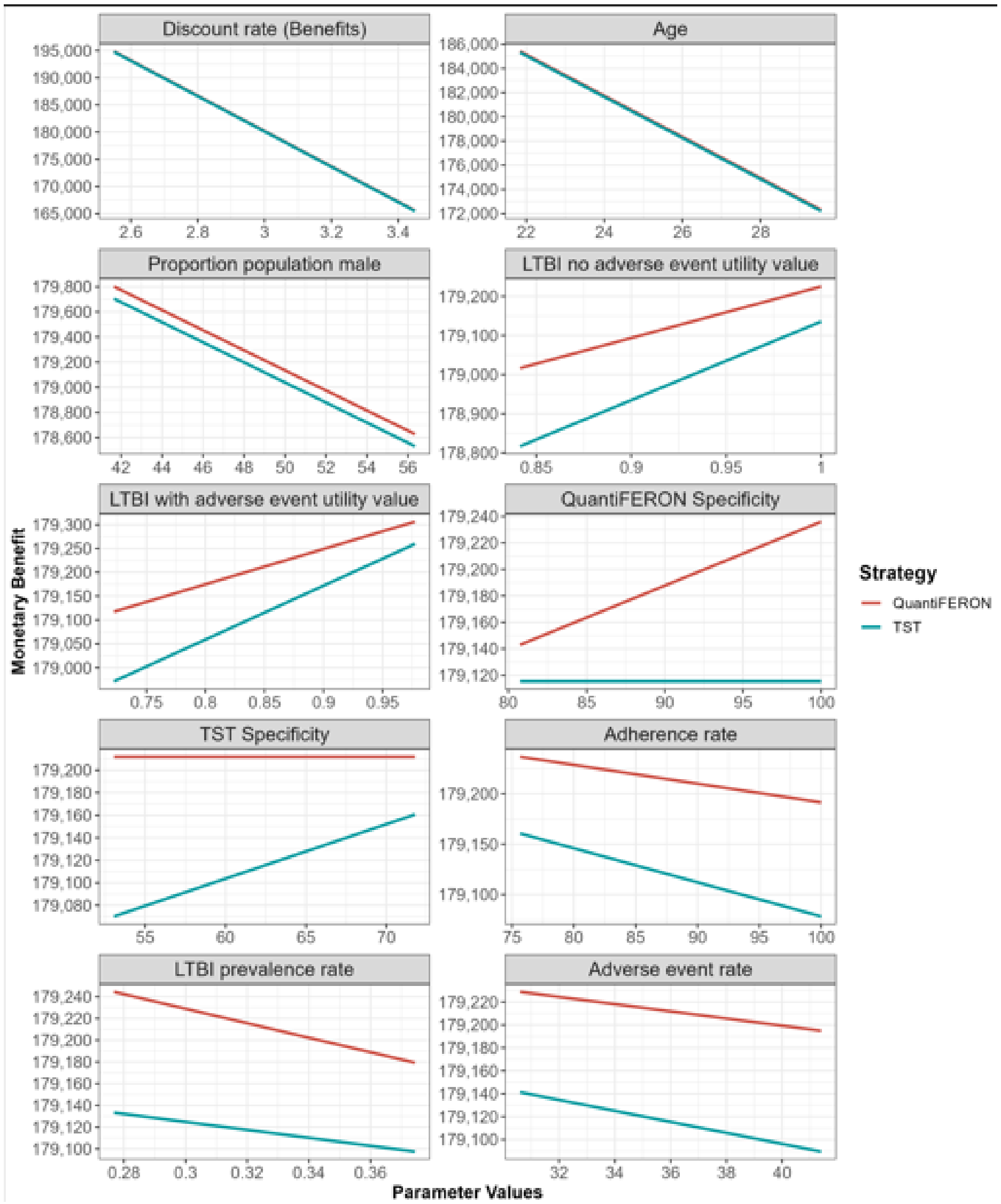

Probabilistic Sensitivity Analysis (PSA)

The PSA indicates that QuantiFERON is more cost-effective than the TST in the majority of simulated scenarios. In the cost-effectiveness plane, red dots representing QFT are mostly clustered in the area of lower costs and higher effectiveness, while the blue dots for TST appear more scattered and tend to be associated with higher costs. Although a few simulations suggest that TST may be slightly less costly in certain situations, the overall trend favours QFT. This is further supported by the large circles representing average outcomes, which clearly show that QFT is, on average, both less expensive and more effective than TST. The cost-effectiveness plane is present in EA10.

Electronic Annex 10. Cost-effectiveness acceptability plane

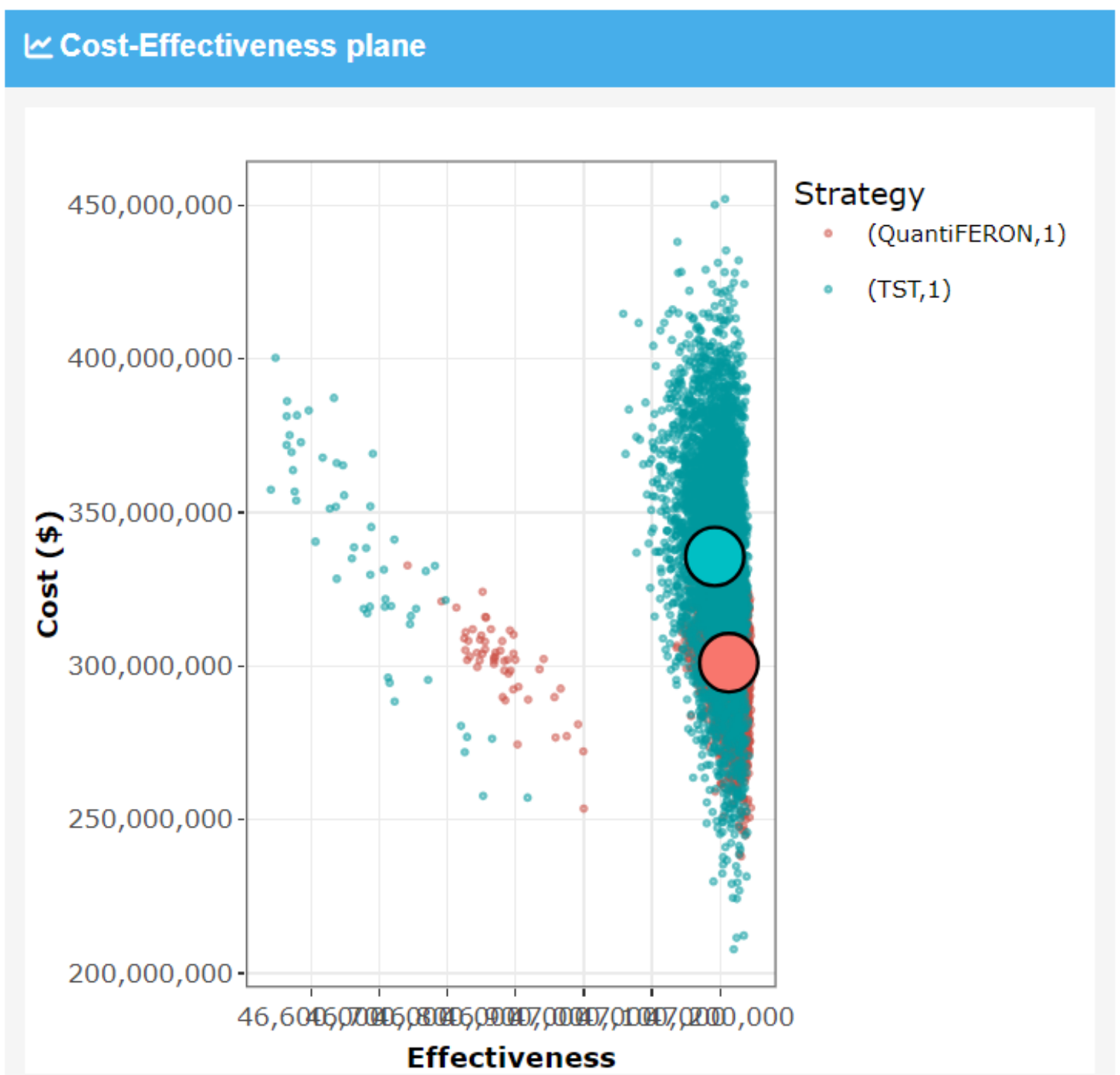

References related to Electronic Annexes

1. World Health Organization. Tuberculosis profile: Philippines. Geneva: WHO.
2. Worldometer. Philippines demographics. Available at: https://www.worldometers.info/demographics/philippines-demographics/
3. Putra IWGAE, et al. The implementation of early detection in tuberculosis contact investigation to improve case finding. J Epidemiol Glob Health. 2019;9(3):191–197.
4. Sia IG, et al. Genotypic characteristics of Mycobacterium tuberculosis isolated from household contacts of tuberculosis patients in the Philippines. BMC Infect Dis. 2013;13:571.
5. Ding C, et al. Prevalence trends of latent tuberculosis infection at the global, regional, and country levels from 1990–2019. Int J Infect Dis. 2022;122:46–62.
6. World Health Organization. Global tuberculosis report 2024. Geneva: WHO, 2024.
7. Chung-Delgado K, et al. Mortality among MDR-TB cases: comparison with drug-susceptible tuberculosis and associated factors. PLoS One. 2015;10(3):e0119332.
8. International Union Against Tuberculosis Committee on Prophylaxis. Efficacy of various durations of isoniazid preventive therapy for tuberculosis: five years of follow-up in the IUAT trial. Bull World Health Organ. 1982;60(4):555–564.
9. Calderon JS, et al. Innovating tuberculosis prevention to achieve universal health coverage in the Philippines. Lancet Reg Health West Pac. 2022;29:100609.
10. World Health Organization. WHO TB Knowledge Sharing Platform: Module 2 – Screening. Available at: https://tbksp.who.int/en/node/626
11. Gupta RK, et al. Discovery and validation of a personalized risk predictor for incident tuberculosis in low transmission settings. Nat. Med. 2020;26:1941–1949.
12. Lofranco VS, et al. Effectiveness and safety of 9-month treatment regimen for multidrug-resistant tuberculosis in the Philippines. J Tuberc Res. 2022;10(2).
13. World Health Organization. Bacillus Calmette-Guérin (BCG) vaccination coverage: Philippines. Geneva: WHO. Available at: https://immunizationdata.who.int/global/wiise-detail-page/bacillus-calmette-gu%C3%A9rin-(bcg)-vaccination-coverage
14. Pai M, et al. Systematic review: T-cell-based assays for the diagnosis of latent tuberculosis infection: An update. Ann. Intern. Med. 2008;149:177–184.
15. Sotgiu G, et al. Tuberculosis treatment and drug regimens. Cold Spring Harb. Perspect. Med. 2015;5:a017822.
16. Dion M-J, et al. Feasibility and reliability of health-related quality of life measurements among tuberculosis patients. Qual. Life Res. 2004;13:653–665.
17. Guo N, et al. Measuring health-related quality of life in tuberculosis: A systematic review. Health Qual. Life Outcomes. 2009;7:1–10.
